# Soluble biomarkers to predict clinical outcomes in non-small cell lung cancer treated by immune checkpoints inhibitors

**DOI:** 10.3389/fimmu.2023.1171649

**Published:** 2023-05-22

**Authors:** Julien Ancel, Valérian Dormoy, Béatrice Nawrocki Raby, Véronique Dalstein, Anne Durlach, Maxime Dewolf, Christine Gilles, Myriam Polette, Gaëtan Deslée

**Affiliations:** ^1^ Inserm UMR-S1250, P3Cell, University of Reims Champagne-Ardenne, SFR CAP-SANTE, Reims, France; ^2^ Department of Respiratory Diseases, Centre Hospitalier Universitaire de Reims, Hôpital Maison Blanche, Reims, France; ^3^ Department of Biopathology, Centre Hospitalier Universitaire de Reims, Hôpital Maison Blanche, Reims, France; ^4^ Laboratory of Tumor and Development Biology, GIGA-Cancer, University of Liège, Liège, Belgium

**Keywords:** liquid biopsy, soluble biomarkers, immunotherapy, non-small cell lung cancer (NSCLC), neutrophil lymphocyte ratio (NLR), circulating tumor (ctDNA), circulating tumor cell (CTC)

## Abstract

Lung cancer remains the first cause of cancer-related death despite many therapeutic innovations, including immune checkpoint inhibitors (ICI). ICI are now well used in daily practice at late metastatic stages and locally advanced stages after a chemo-radiation. ICI are also emerging in the peri-operative context. However, all patients do not benefit from ICI and even suffer from additional immune side effects. A current challenge remains to identify patients eligible for ICI and benefiting from these drugs. Currently, the prediction of ICI response is only supported by Programmed death-ligand 1 (PD-L1) tumor expression with perfectible results and limitations inherent to tumor-biopsy specimen analysis. Here, we reviewed alternative markers based on liquid biopsy and focused on the most promising biomarkers to modify clinical practice, including non-tumoral blood cell count such as absolute neutrophil counts, platelet to lymphocyte ratio, neutrophil to lymphocyte ratio, and derived neutrophil to lymphocyte ratio. We also discussed soluble-derived immune checkpoint-related products such as sPD-L1, circulating tumor cells (detection, count, and marker expression), and circulating tumor DNA-related products. Finally, we explored perspectives for liquid biopsies in the immune landscape and discussed how they could be implemented into lung cancer management with a potential biological–driven decision.

## 1 Introduction

Lung cancer represents the first cause of cancer‐related deaths worldwide with over 1.5 million deaths in 2018 and an incidence superior to 2 million (11.6%), largely represented by non-small cell lung cancer (NSCLC) (1). Lung cancer is diagnosed at a locally advanced or metastatic stage in most cases, leading to no curative options and poor outcomes (2). In recent decades, many innovative strategies have been designed, namely tyrosine kinase inhibitors (TKIs) targeting oncogenic drivers or immunotherapies (3). On the one hand, personalized medicine based on molecular targetable alterations has emerged from proof of concept to current clinical applications with restricted indications to a sub-population (4). On the other hand, immune checkpoint inhibitors (ICI) are now largely employed but obtain various response rates with fewer than 40% of responders among a population selected on programmed death-ligand 1 (PD-L1) expression (5).

Many biomarkers have been investigated through the last decades to improve clinical cancer management and patient outcomes. First, biomarkers designed to predict better, and longer responses have been proposed, such as PD-L1. PD-L1 expression in tumor biopsy is the strategy that allows identifying a subpopulation of patients benefiting from ICI. For example, patients with a high PD-L1 tumor proportion score (TPS ≥ 50%) benefit from ICI in first-line (vs platinum-based chemotherapy) (6–8). However, resistance and relapse fatally occur in most cases. Consequently, global age-standardized 5-year survival remains within the range of 10-20% and a limited increase of up to 5% has been observed (9), arguing the need to further refine and improve clinical lung cancer management. Therefore, other approaches have been explored in plasma or total blood. Soluble biomarkers have the advantages to allow real-time monitoring, repeatable, and easily feasible at every step of lung cancer (from the diagnosis throughout the progression of the disease) including non-evaluable radiographic diseases, named biological minimal residual diseases (MRD) (10). Liquid biopsy is now even integrated into clinical practice to research and/or monitor oncogenic addiction under TKI treatment (11). Circulating tumor-derived products are various and offer wide potential applications, especially in the ICI field (12). Inflammation-related biomarkers are particularly promising since inflammation is associated with a worse prognosis in solid tumors due to its effect on the immune modulation, into both tumor cells and its microenvironment, influencing disease-related outcomes (13). These biomarkers include immunoregulatory cells, soluble mediators, and a panel of features including absolute neutrophil, eosinophil, lymphocyte counts, or ratios (14). To date, no soluble biomarker has yet been approved and validated for the management of lung cancer patients, despite important recent technical advances. In this context, there is an emerging interest to identify one to predict ICI benefit, overcoming limitations due to tissue-based analysis (15).

**Figure 1:**
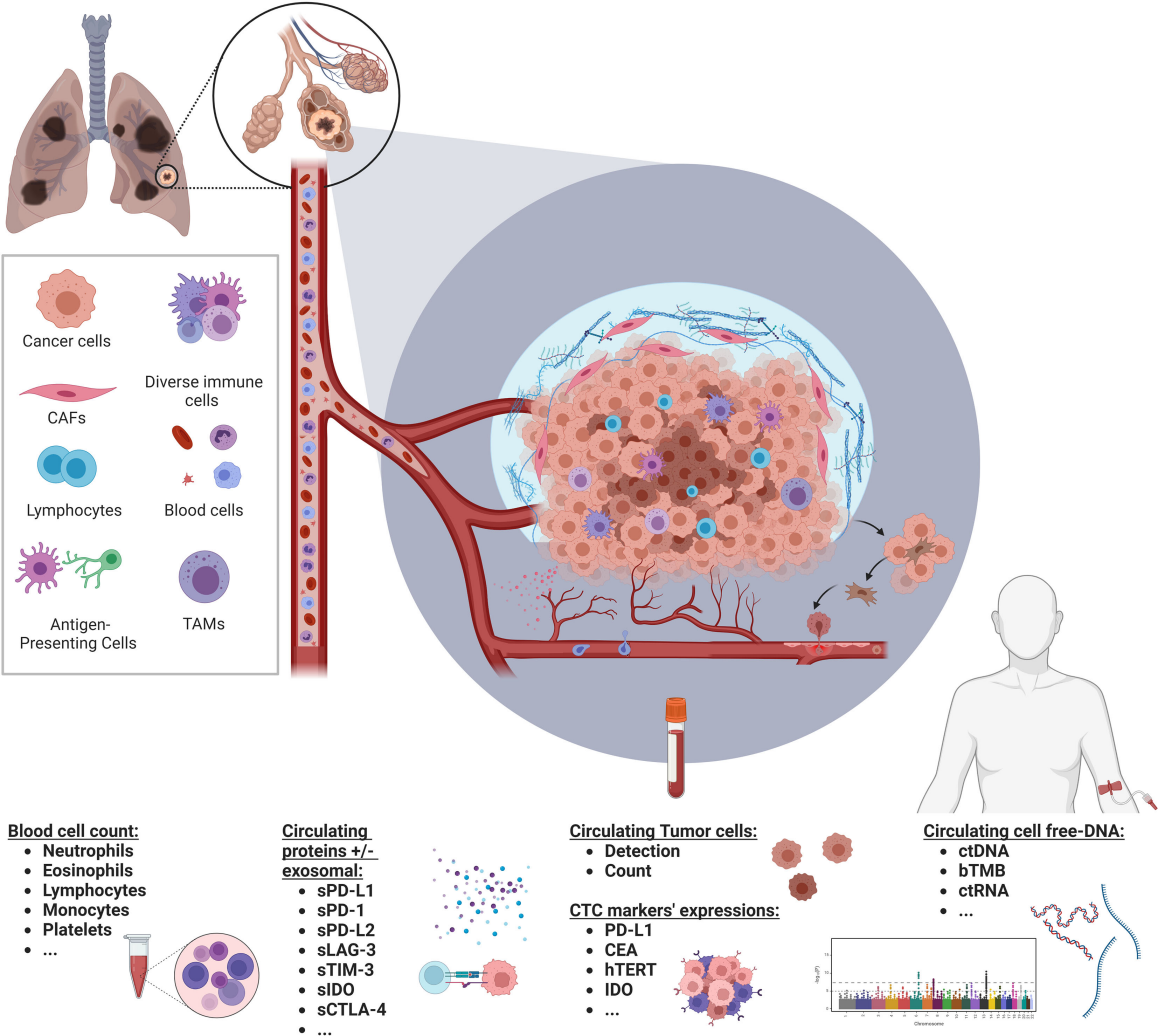
Soluble biomarkers in the immune landscape of NSCLC. Graphical abstract illustrating various types of soluble biomarkers with potential clinical relevance in the immunotherapy field in a non-small cell lung cancer context. Created with BioRender.com.

Numerous serum-based biomarkers have already been explored or are currently under investigation. Among the most promising, the blood cell count of neutrophils, lymphocytes, and platelets have been associated with ICI efficacy with potential prognostic value (15). Other promising serum-based biomarkers include soluble PD-L1 (sPD-L1) (16), circulating tumor cells (CTCs) (17), blood tumor mutational burden (bTMB), or circulating tumor DNA (ctDNA) (18). **Figure 1** – Graphical Abstract.

In this review, we discuss the potential relevance of such soluble biomarkers associated with clinical outcomes in NSCLC treated by ICI. We also analyzed their limitations precluding their implementation into clinical management. Finally, we selected active clinical trials exploring soluble biomarkers in NSCLC treated by ICI focusing on how their results could be integrated into clinical practice.

## 2 Non-tumoral blood cell count

Systemic inflammation is a well-known related condition impacting tumor responses under ICI treatment in solid cancers (19). Cytokine profiles are thus modified in case of an inflammatory tumor with high IL-6 and TNF-α levels, affecting myelopoiesis (20), and resulting in a shift in blood-cell numerations. Neutrophils and other immune cells such as lymphocytes, platelets myeloid-derived suppressor cells, and monocytes also secrete proinflammatory cytokines (i.e. VEGF, IL-6/8, or TGF-beta) (21). Considering that these circulating immune cells also represent a broad part of ICI therapy effectors, immune cell count was evaluated in serum to predict ICI efficacy. Here under we review the impact of neutrophils, lymphocytes, and platelets, and their variation (Δ) in the case of longitudinal monitoring of clinical outcomes under ICI treatment.

### 2.1 Absolute neutrophils count

Absolute neutrophil count (ANC), as a predictor for ICI response, has been investigated in many studies. The largest included 213 patients, retrospectively comparing biological profiles between long-term responders and non-long-term responders (22). Δ ANC decreased at 4 weeks and was associated with longer responses (p=0.018). In another large retrospective cohort of 191 patients, lower ANC at baseline was associated with better OS (p=0.048) with similar observations at first re-evaluation (23). All other studies were concordant with these results, despite ICI heterogeneity and thresholds. The principal ANC cut-off was 6.0 10^3^/µL, whereas some authors proposed higher values until 7.5 10^3^/µL (24). The main limitation for ANC integration in current practice to predict ICI outcomes remain a restricted number of studies. Moreover, ICI combined with chemotherapy could involve G-CSF stimulation, hardening Δ ANC interpretation in this context.

### 2.2 Absolute eosinophils count

Absolute eosinophil count (AEC) was investigated in a few studies. The largest enrolled 191 patients, mostly treated with Nivolumab (n=100) and Pembrolizumab (n=58) (23). Interestingly, the authors reported an induced early increase in AEC, more frequently in responding patients, independently of PD-L1 and immune-related adverse events (p<0.001). Other studies tended to be negative regarding AEC as a predictor of ICI response. Otherwise, many parameters could influence AEC such as corticosteroids in premedication (for chemotherapy combination or palliative radiotherapy support). The interest of AEC thus appears limited for further studies to predict ICI response.

### 2.3 Absolute lymphocyte count

Absolute lymphocyte count (ALC) was extensively assessed, broadly co-evaluating lymphocyte ratio as described in the following sections. Murakami et al. conducted the largest study, with 213 patients, all treated with Nivolumab (22). ALC was not associated with ICI outcomes. Two other studies equally dimensioned with 203 and 191 patients respectively, were consistent despite various anti-PD-1 and anti-PD-L1 drugs: ALC did not differ according to ICI response (23, 25).

### 2.4 Circulating immune-suppressive cells

Flow cytometry (FC) allows a deep analysis of peripheral blood cell subpopulations such as myeloid-derived suppressor cells (MDSCs) and their sub-populations including monocytic myeloid-derived suppressor cells (M-MDSCs) and granulocytic MDSC (G-MDSCs) (26). Both tissular MDSCs and circulating subset play an important immunosuppressive role and negative effect on ICI in animal tumor models (27). Due to their immunosuppressive effects, MDSCs exhibit protumoral effects and associate with poorer prognosis. Thus, Bronte et al. reported in a meta-analysis pooling 14 studies (905 NSCLC patients) that high level of circulating M-MDSCs was associated with short PFS (HR=2.67, p<0.0001) and OS (HR=2.10, p<0.0001) (28). The proof of concept was established in a melanoma context treated by ICI (i.e ipilimumab) with a benefit from ICI in patients with low frequencies of M-MDSCs (29). In NSCLC, various reports are available in the ICI context. Feng et al. observed a rapid increase in NK cell fraction in 27 NSCLC patients responding to nivolumab, along with a reduction of G-MDSCs (30). Similar results were reported in a cohort of 132 NSCLC patients treated by anti-PD-1 therapy: lower levels of circulating M‐MDSCs, polymorphonuclear (PMN)‐MDSCs, and CD39+CD8+ T cells at baseline were associated with longer PFS and OS (31). In the same report, PD-L1 TPS was not correlated with the proportions of suppressive immune cells, including PMN-MDSCs and M-MDSCs, or with the clinical outcome. This was concordant with another report based on 22 NSCLC patients: patients with M-MDSC values upper than the median experienced shorter PFS (HR=2.51, p=0.046) and OS (HR=2.68, p=0.042) (32). FC could both assess pro and anti-tumor cell subsets such as regulatory T cells and MDSCs. Kim et al. thus proposed that the ratio between peripheral regulatory T cells to lox-1+ PMN MDSCs could predict the early response to ICI in NSCLC patients (33). Similarly, Youn et al. reported in 62 NSCLC patients that the NK cell-to-Lox-1+ PMN-MDSC ratio was significantly higher in patients benefiting from ICI (p<0.0001) (34). FC thus allows to standardize evaluation of immune cell subsets and potentially predicts clinical outcome for NSCLC patients treated by ICI. FC has become invaluable for biomarker research, providing detailed information on single cells in a heterogeneous population. However, only few clinical trials investigated FC interest in ICI context. Its validation and relevance at larger scale need to be further investigated.

## 3 Non-tumoral blood cell ratios

As previously reviewed, the absolute count for circulating non-tumoral cells is not sufficient to predict clinical outcomes under the ICI regimen. Also, the markers of the systemic inflammatory response (such as plasma C-reactive protein (CRP) or hypoalbuminemia) have been shown to play a major role in cancer progression and aggressiveness (35). Many systemic inflammatory markers have thus been reported as prognostics markers in NSCLC, mainly based on the cell count ratio between two or more non-tumoral cell subsets. Among them, platelet-to-lymphocyte ratio (PLR), neutrophil-to-lymphocyte ratio (NLR), and lymphocyte-to-monocyte ratio (LMR) have been previously proposed as prognostic markers (36–38). Moreover, the ratio between various subsets of cells could reflect the systemic inflammation with a lower intra- and inter-individual variation, especially during course of the treatment. As an additional hypothesis, the ratio between pro and anti-tumoral factors could also introduce precision and robustness to predict tumor immune sensibility. We review here the impact of neutrophils, lymphocytes, and platelets respective ratios, and their variation (Δ) for NSCLC patients treated by ICI.

### 3.1 Platelet to lymphocyte ratio

Platelet to lymphocyte ratio (PLR) is a common ratio combining two parameters related to chronic inflammation. We reviewed 16 studies exploring PLR as a potential predictor of ICI response and survival. Ksienski’s report was the largest study published, retrospectively investigating PLR to predict ICI outcomes in 220 patients with NSCLC treated by Pembrolizumab in the frontline for 95% of them (39). In this study, patients with high PLR at baseline had worse OS (median: 4.0 vs 15.4 months, HR: 2.03, p=0.006), suggesting that PLR could predict ICI response and benefit. Interestingly, this study included only patients with high PD-L1 expression (TPS >50%) and thus highlighted that PLR may be independent of PD-L1 expression to predict ICI benefit. A recent meta-analysis published by Liu et al. integrated 15 studies focused on PLR in the ICI field of NSCLC (40). In this meta-analysis, the authors established that high PLR was associated with worse OS (HR:  1.49, p<0.001, I2  = 57.6%, p=0.003), driven by worse PFS (HR = 1.62, p<0 .001). To note, the I2 index reflects the degree of heterogeneity in a meta-analysis among included studies with higher heterogeneity for higher I2 indexes. Very interestingly, there was no association between PLR and OS in the group ≥ 200 when stratified by cut-off point (HR: 1.35, p=0.172). This discrepancy illustrates the heterogeneity in studies assessing PLR as a predictor of ICI response: the range for PLR cut-off varies from 144 to 441 and introduces a broad bias.

### 3.2 Systemic immune inflammation index

Another composite score based on three types of blood cell was also developed: the systemic immune inflammation index (SII) (SII = Platelet × Neutrophils/Lymphocytes counts). This index was first developed in the context of hepatocellular carcinoma (41). Larger studies then proposed that higher SII could predict worse clinical outcomes in various solid cancers, including NSCLC in terms of PFS, OS, and disease-free survival (DFS) (42, 43).

Only three studies investigated SII interest in NSCLC patients treated by ICI. One retrospective study of 44 patients treated by nivolumab monotherapy in the second line reported a significant association: low SII at baseline (<603.5) predicted longer PFS (HR=0.34, p=0.006) and OS (HR=0.16, p=0.005) and remained significant in a multivariate analysis (44). These results remain controversial, without association between SII at baseline (cut-off value of 730 and 792.07, respectively) and tumor response in two other studies (45, 46). Interestingly, these two studies both reported a significant prediction for dynamic change of SII throughout the ICI course of 6 weeks. Indeed, Fang et al. reported a shorter PFS for patients with an increased in SII from baseline (HR=1.731, p=0.027). Similarly, Jin Suh et al. observed a worse PFS for patients with a post-treatment SII ≥ 730 at 6 weeks (median: 2.8 vs 8.1 months, p=0.033). In earlier stages (stages I-IIIB), the same observations were done for NSCLC patients treated by chemoimmunotherapy: SII at baseline did not predict pathological response. However, on-treatment SII and a decrease of SII from baseline exhibited more frequently a major pathological response (p<0.01) (47).

SII predictive capacity was not specific to the ICI context, since similar results have been reported in EGFR mutant patients treated by TKIs (48–50). Considering inconsistent and non-specific results, SII is currently set aside to predict clinical issues for patients treated by ICI.

### 3.3 Lymphocyte to monocytes ratio

A few studies investigated lymphocyte-to-monocyte ratio (LMR) to predict ICI response. The largest one was a retrospective cohort of 262 patients mainly treated with Nivolumab (n=131) or Pembrolizumab (n=95). The patients with LMR < 2.12 at baseline were exposed to shorter OS (HR: 1.62, p=0.02) in multivariate analysis (51). In other available studies, low LMR was also associated with worse outcomes for patients with NSCLC under ICI regimens. These parameters remain rarely explored. A meta-analysis integrated 4 studies investigating this parameter: patients with low LMR had worse OS without heterogeneity (HR = 0.45, p <0 .001) (40). Finally, only one study compared PD-L1 and LMR abilities to predict ICI benefits with similar results. In Katayama et al. ‘s report, PD-L1 TPS was not significantly predictive for OS or PFS, while LMR > 1.5 was associated with better PFS and OS (HR: 0.418, p=0.004 and HR: 0.30, p<0.0001, respectively) (52). Of note and as a main limitation, LMR relevant cut-off was not consensual ranging from 1.5 to 2.12. This critical point needs to be elucidated in larger studies.

### 3.4 Prognostic nutritional index

Nutrition and immune features share a close relationship and can both modify tumor aggressivity and prognosis in cancer patients (53). The nutritional status, the muscle mass, and the inflammatory status could reflect cancer-related cachexia and impact the immune system, leading to potential ineffective ICI (54). The prognostic nutritional index (PNI) is calculated as follows: [(10 × serum albumin (g/dL)) + (0.005 × total lymphocyte count)]. It is an efficient indicator for assessing the nutritional and immunological conditions of cancer patients. The parameters are routinely assessed in laboratory tests during clinical cancer management and are easily repeated. Numerous studies reported an association between baseline PNI and survival in various cancers, including NSCLC (55, 56). For example, PNI predicted both early-progression (OR=3.709, p=0.011) and shorter OS (HR= 7.596, p<0.001) for patients with lower PNI (significant cut-off value determined by ROC curve) (57). A meta-analysis dedicated to PNI included 12 studies, enrolling 13590 NSCLC patients treated by ICI (58). The Cut-off value for PNI ranged from 31.1 to 48. The findings demonstrated that patients treated by ICI with low PNI at baseline had both shorter OS (HR=2.24, 95% CI=1.57–3.20) and PFS (HR=1.61, 95% CI=1.37–1.88). Mahiat C. et al. explored systemic inflammation/nutritional status (including PNI) as predictive factors in 3 metastatic NSCLC cohorts treated in the first line by ICI monotherapy (n=75), ICI combined with chemotherapy (n=56), or chemotherapy alone (n=221). Their results supported that systemic inflammation/nutritional status could be associated with the outcomes independently of the treatment, and were therefore prognostic but not predictive (59). The ICI efficacy predicted by PNI also seemed independent of PD-L1 expression, since no association between PD-L1 TPS and PFS/OS was reported while a lower PNI was significantly associated with shorter PFS (HR: 1.704, p<0.05) (60). Consistent comparisons were reported in external NSCLC cohorts (57, 61). The non-specific prognosis was also supported by Sheng et al.: low PNI at baseline was predictive of worse survival in the EGFR mutated context (untreated by ICI) (62). Low levels of baseline PNI could thus be a significant predictor of worse clinical outcomes for patients treated with ICIs. However, its specificity with ICI and relevant cut-off remains unclear and needs to be assessed in further prospective and larger cohorts.

### 3.5 Neutrophil to lymphocyte ratio and derived neutrophil to lymphocyte ratio

Neutrophil to lymphocyte ratio (NLR) and derived neutrophil to lymphocyte ratio (dNLR) were the broadest investigated features based on blood cell count. NLR assessed by absolute neutrophil count divided by absolute lymphocyte count is the most explored parameter as a potential predictor of clinical outcomes for NSCLC patients treated by ICI. NLR is a marker for the general immune response to various stress conditions (63, 64).

These studies are reviewed in **Table 1**. The most relevant and robust trial was LIPS-3, aiming to stratify the prognosis of patients treated by ICI. It retrospectively included 784 patients (201 in a training group and 583 in a validation group), all of them treated with Pembrolizumab in the frontline, thus with a TPS of PD-L1 ≥ 50% (85). Based on a threshold of 4, low NLR was associated with better OS in both cohorts, reaching 76.6% at 1 year. Interestingly, the authors proposed combining NLR with other factors such as PS-ECOG and corticosteroid pre-treatment to improve their prognostic score. As reviewed, a very large part of studies exploring NLR are consistent: low NLR at baseline was associated with better responses and clinical outcomes under an ICI regimen. However, patients and ICI are very heterogeneous, considering the line of pre-treatment, PS-ECOG, sub-type histology (squamous vs non-squamous), PD-L1 expression, or combination with chemotherapy. A recent meta-analysis aggregated 31 studies (40): high NLR was associated with shorter OS (HR 2.13, p<0.001) with significant heterogeneity (I2 = 83.8%, p<0 .001). Very similar results were observed for Δ NLR: increased NLR through ICI administration was associated with worse survival (HR = 1.77, p < 0.001, I2 = 79.5%, p <0.001). The sub-group analysis performed on the cut-off showed a significant association for the NLR threshold of 5 (HR=1.94, p<0.001), which remains the most employed cut-off, with a range from 2.8 to 5. Some studies compared and adjusted NLR levels with PD-L1 expression in multivariate analysis predicting PFS or OS for patients treated by ICI. Most of them were consistent, observing an independence between NLR and PD-L1 expression (51, 83, 95).

**Table 1:** Non-tumoral blood cell count biomarkers.

Study	n	Biomarker	ICI used	Time of assessment	Conclusion	Design	Respective cut-off (If significant)	Comment
(Karantanos et al., 2019 (65))	22	ALC ANC	Nivo	Baseline and on-treatment	ALC at baseline and 6 w positively correlated with OS (p<0.01) ANC/ALC at baseline was negatively associated with OS (p<0.05)	Retro	ALC ≥ 0.9, 1.3 and 1.7 10^3^/µL	Previous radiation was associated with higher ANC and lower ALC
(Diem et al., 2017 (66))	52	ALC ANC NLR PLR	Nivo	Baseline	Pts with high NLR were associated with worse OS (HR: 3.3, p<0.013) and lower ORR Pts with high PLR were associated with worse OS (HR: 4.1, p<0.001) and lower ORR		NLR ≥ 5 PLR ≥ 262	No significant association between PFS with both NLR and PLR
(Khunger et al., 2018 (67))	109	ALC ANC AMC NLR Δ NLR	Nivo	Baseline and on-treatment	Post-treatment NLR ≥ 5 after 2 cycles of Nivo was associated with poor OS (median: 29.1 mths *vs* 24.2 mths, p<0.001) ΔNLR > 0 after 2 cycles of Nivo was associated with non-responders (p=0.027)	Retro	NLR ≥ 5 Quartiles for ALC and ANC ΔNLR ≥ 0	Pts in the highest quartile of post-treatment ALC had superior OS compared to the remaining population (log-rank p=0.0113) Pts in the highest quartile of post-treatment ANC had inferior OS compared to all others (log-rank p=0.0027)
(Facchinetti et al., 2018 (68))	54	ANC WBC NLR	Nivo	Baseline	Pts with higher WBC (p=0.004), ANC (p=0.004) and NLR (p=0.001) had poorer OS	Prosp	WBC ≥ 8.8 10^3^/µL ANC ≥ 6 10^3^/µL NLR ≥ 4	
(Patil et al., 2017 (69))	115	ANC AMC NLR Δ NLR	Nivo	Baseline and on-treatment	ANC, AMC, and NLR were associated with worse OS (HR: 1.17, p=0.00001; HR 4.53, p=0.04 and HR: 1.09, p=0.0002)	Prosp	ANC ≥ 6 AMC ≥ 0.5 NLR ≥ 2.8 ΔNLR ≥ 0	ΔNLR > 0 was associated with non-responders (p=0.03)
(Park et al., 2018 (70))	159	ANC ALC NLR AEC PLR Δ NLR	Nivo	Baseline and on-treatment	Pts with high NLR had worse PFS (HR: 1.68, p=0.015) ΔNLR did not significantly correlate with PFS Median PFS for the iSEND good, intermediate, and poor were 17.4, 5.3, and 2.8 mths, respectively (p<0.0001)	Retro	Composite iSEND score including NLR ≥ 5 and ΔNLR ≥ 0	
(Daher et al., 2021 (71))	108	ANC WBC NLR dNLR PLR	Nivo	Baseline	Pts with high dNLR correlated significantly with worse OS (HR: 1.12, p<0.05)	Retro	dNLR ≥ 3	Only ΔNLR remained significant in multivariate analysis
(Pu et al., 2021 (24))	184	ALC ANC AMC NLR PLR	Pembro, n=98 Nivo, n=86	Baseline	Pts with high NLR or PLR had independent poor OS (HR: 1.964, p<0.05; HR: 4.255, p<0.001, respectively)	Retro	ANC ≥ 7.5 10^3^/µL NLR ≥ 5 PLR ≥ 200 ALC ≥ 1.0 10^3^/µL	
(Murakami et al., 2022 (22))	213	ANC ALC NLR dNLR Δ NLR	Nivo	Baseline and on-treatment	Pts with Δ NLR ≤ 1 were associated with longer OS (HR: 3.97, p<0.05) in multivariate analysis	Retro	Δ NLR ≥ 1	
(Sibille et al., 2021 (23))	191	ALC ANC NLR PLR AEC	Nivo, n=100 Pembro, n=58 Durva, n=22 Atezo, n=11	Baseline	A lower baseline ANC correlated with longer OS (p = 0.049) At 1^st^ evaluation, high ANC and NLR correlated with worse OS (p<0.05)	Retro	Not provided	
(Soyano et al., 2017 (72))	52	ANC ALC NLR AEC	Nivo, n=48 Pembro, n=4	Baseline	Pts with high ANC had a worse OS (HR: 2.46, p=0.025) and PFS (HR: 2.41, P=0.009) Pts with both high ANC/ALC had worse OS (HR: 2.41, p=0.027) and PFS (HR: 2.08, p=0.027)	Retro	ANC ≥ 6.06 10^3^/µL	
(Bagley et al., 2017 (73))	175	NLR PLR	Nivo	Baseline	Pts with high NLR were independently associated with worse OS (median: 5.5 mths *vs* 8.4 mths; HR: 2.07; p=0.002) and PFS (median: 1.9 mths *v*s 2.8 mths; HR: 1.43, p=0.04)	Retro	NLR ≥ 5	
(Suh et al., 2018 (46))	54	NLR Δ NLR	Nivo	Baseline and on-treatment	Pts with high post-treatment NLR had significantly shorter PFS (median: 1.3 mths *vs* 6.1 mths, p<0.001)	Retro	NLR ≥ 5 PLR ≥ 169 ΔNLR ≥ 0	Baseline NLR, PLR, and SII were not predictive of response
(Rogado et al., 2017 (74))	40	NLR NCP	Nivo	Baseline	NLR < 5 and NCP < 80% were associated with improved PFS (HR:6.7, p<0.001 and HR: 0.09, p<0.001) and OS (HR: 4.4, p<0.001 and HR: 0.2, p=0.02)	Retro	NLR ≥ 5 NCP ≥ 80%	Comparable results in the CT group
(Liu et al., 2019 (44))	44	NLR PLR SII	Nivo	Baseline	Low SII, NLR, and PLR were associated with better PFS (HR: 0.34, p=0.006; HR: 0.46, p=0.048 and HR: 0.39, p=0.025, respectively) and OS (HR: 0.16, p=0.005; HR: 0.20, p=0.002 and HR: 0.20, p=0.008)	Retro	SII ≥ 603.5 NLR ≥ 3.07 PLR ≥ 144	
(Shiroyama et al., 2018 (75))	201	NLR	Nivo	Baseline	Pts with high NLR had worse PFS (median: 1.5 mths *vs* 3.5 mths, p=0.019)	Retro	NLR ≥ 4	
(Fukui et al., 2019 (76))	52	NLR	Nivo	Baseline	Pts with high baseline NLR had poorer OS (HR: 4.52, p=0.013)	Prosp	NLR ≥ 5	
(Passiglia et al., 2019 (77))	45	NLR Δ NLR	Nivo	Baseline and on-treatment	Pts with increased NLR ≥ 20% at 6 w had significantly worse survival outcomes (median OS: 8.7 mths *vs* 14.6 mths, p=0.035; median PFS: 5.2 mths *vs* 10.3 mths, p=0.039)	Retro	ΔNLR ≥ 20%	
(Russo et al., 2018 (78))	62	dNLR PLR	Nivo	Baseline	PFS and OS did not differ according to dNLR for pts treated by Nivo	Retro	ANC ≥ 7.5 10^3^/µL dNLR ≥ 3 PLR ≥ 160 PLT ≥ 450	Control CT group
(Takeda et al., 2018 (79))	30	NLR Δ NLR PLR	Nivo	Baseline and on-treatment	Pts with high NLR at 4 w were associated with shorter PFS (HR 5.995, p<0.05)	Retro	NLR ≥ 5 ΔNLR ≥ 0 PLR ≥ 150	
(Amaral et al., 2019 (80))	32	NLR PLR	Nivo, n=20 Pembro, n=12	Baseline	High NLR or PLR above the mean were independent predictive factors for worse PFS (11 mths *vs* 6 mths, HR 3.33, p=0.056 and 12 mths *vs* 6 mths, HR: 3.9, p=0.025, respectively)	Retro	Not provided	
(Dusselier et al., 2019 (81))	59	Δ NLR	Nivo	Baseline and on-treatment	Δ NLR < 1 prolonged OS (HR: 0.001, p=0.001) and remained significant in multivariate analysis (HR:0.12, p=0.001)	Retro	Δ NLR ≥ 1 NLR ≥ 5 PLR ≥ 262	Baseline NLR and PLR were not predictive of response
(Ren et al., 2019 (82))	147	NLR	Nivo, n = 60 Pembro, n= 87	Baseline	Pts with low NLR had better OS (p=0.009) and PFS (p=0.017).	Retro	NLR ≥ 2.5	
(Pavan et al., 2019 (83))	184	NLR PLR	Nivo, n= 145 Pembro, n= 34 Atezo, n=7	Baseline	Pts with low NLR had better PFS (median: 7.4 mths *vs* 3.1 mths, p=0.003) and OS (HR: 0.468, p=0.001)	Retro	NLR ≥ 3 PLR ≥ 180	
(Banna et al., 2020 (84))	132	NLR	Pembro	Baseline	Pts with low NLR had better PFS (12.0 mths *vs* 5.7 mths, p=0.01) and OS (HR: 0.45, p=0.005)	Retro	NLR ≥ 5	
(Banna et al., 2021 (85))	784	NLR	Pembro	Baseline	Pts with low NLR had better PFS (HR: 2.29, p<0.001)	Retro	NLR ≥ 4	
(Banna et al., 2022 (86))	128	NLR	Pembro	Baseline	Pts with low NLR had better PFS (median: 51 mths *vs* 1.8 mths, HR:1.9, p=0.005)	Retro	NLR ≥ 4	Only PS2+ pts
(Ksienski et al., 2021 (39))	220	NLR PLR	Pembro	Baseline	Pts with high NLR (HR: 2.31, p<0.0001) or PLR (HR: 2.03, p=0.006) had worse OS	Retro	NLR ≥ 6.4 PLR ≥ 441.8	
(Peng et al., 2020 (87))	102	NLR	Nivo, n=11 Pembro, n=26 Toripa, n=30 Sinti, n=35	Baseline	Pts with high NLR had worse outcomes according to PFS (p=0.049) and OS (p=0.007)	Retro	NLR ≥ 5	
(Ayers et al., 2021 (88))	173	NLR Δ NLR	Nivo or Pembro	Baseline and on-treatment	Pts with high NLR had worse OS (HR: 1.66, p=0.019) Pts with ΔNLR > 1 also had worse OS (HR: 3.33, p<0.0001)	Retro	NLR ≥ 5 ΔNLR ≥ 1	
(Takeyasu et al., 2021 (89))	145	NLR	Pembro	Baseline	Not significate	Retro	NLR ≥ 5	Only TPS > 50%
(Russo et al., 2020 (90))	187	NLR PLR	Nivo	Baseline	Pts with low NLR had a better PFS (HR: 0.64, p<0.05) and OS (HR: 0.48; p=0.001) Pts with low PLR had better PFS (HR: 0.67; p<0.05) and OS (HR: 0.66; p=0.05)	Retro	NLR ≥ 5 PLR ≥ 200	
(Takada et al., 2020 (51))	226	dNLR LMR	Nivo, n=131 Pembro, n = 95	Baseline	Pts with high dNLR had worse PFS (HR: 1.56, p<0.05) and OS (HR: 1.68, p<0.05) Pts with low LMR at baseline had worse OS (HR: 1.62, p=0.02)	Retro	dNLR ≥ 2.79 LMR ≥ 2.12	NLR was only significant in univariate analysis
(Alessi et al., 2021 (91))	221	dNLR	Pembro	Baseline	Pts with low dNLR had better PFS (HR: 0.47, p<0.001) and OS (HR: 0.32, p<0.001)	Retro	dNLR ≥ 2.6	
(Yuan et al., 2021 (25))	203	ALC dNLR Δ NLR	Nivo, n=43 Pembro, n=50 Camre, n=31 Toripa, n=26 Sinti, n=31 Tisleli, n=22	Baseline and on-treatment	Pts with high dNLR were associated with poorer OS (HR: 1.434, p=0.035) in multivariate analysis	Retro	dNLR ≥ 2.35	Control CT group
(Chen et al., 2021 (92))	101	NLR Δ NLR	Nivo, n=49 Pembro, n=47 Camre, n=1 Toripa, n=3 Sinti, n=1	Baseline and on-treatment	Patients with either high NLR or positive Δ NLR showed worse OS (HR: 3.12, p<0.001) and PFS (HR: 3.45, p<0.001)	Retro	NLR ≥ 4.5 ΔNLR ≥ 0	Only PS2+ pts
(Lim et al., 2021 (93))	89	NLR dNLR Δ NLR Δ dNLR	Nivo, n=33 Pembro, n=56	Baseline and on-treatment	Pts with increased NLR had worse PFS (median: 2.6 mths *vs* 9.5 mths, p<0.001) Pts with increased dNLR showed worse PFS (median: 4.2 mths *vs* 9.2 mths, p=0.001)	Retro	ΔNLR ≥ 1 Δ dNLR ≥ 1	
(Jiang et al., 2020 (94))	76	PLR Δ NLR	Nivo, n=59 Durva, n=17	Baseline and on-treatment	Pts with high PLR had a poorer PFS (HR: 3.15, p=0.006) and OS (HR: 3.26, p=0.014)	Retro	PLR > 168.13	
(Petrova et al., 2020 (95))	119	PLR NLR Δ NLR	Pembro	Baseline and on-treatment	Pts with high NLR at baseline showed significantly shorter PFS (median: 6.86 mths, p<0.001)	Retro	PLR > 200 ΔNLR ≥ 25%	
(Xiong et al., 2021 (96))	41	PLR NLR Δ NLR	Nivo, n=19 Pembro, n=19 Atezo, n=2 Toripa, n=1	Baseline and on-treatment	Both PLR and NLR at baseline were not a predictor for PFS Pts with high NLR at 6w had shorter PFS (HR: 0.29, p=0.04)	Retro	NLR ≥ 5 PLR > 169	
(Katayama et al., 2020 (52))	81	PLR NLR LMR	Atezo	Baseline	Pts with high NLR, low LMR, and/or high PLR at baseline had shorter PFS and OS (HR: 3.78; 0.3 and 2.82, respectively, p<0.001)	Retro	NLR ≥ 5 PLR > 262 LMR ≥ 1.5	
(Matsubara et al., 2020 (97))	24	PLR NLR	Atezo	Baseline	Pts with high NLR had worse OS (HR: 3.53, p=0.038) in multivariate analysis PLR was not significant for OS prediction	Retro	NLR ≥ 5 PLR > 150	
(Rossi et al., 2020 (98))	65	NLR LMR Δ NLR	Nivo	Baseline and on-treatment	In multivariate analysis, only an increased NLR was associated with shorter OS (p<0.0001)	Retro	NLR ≥ 4.9 ΔNLR > 0 LMR ≥ 1.38	
(Simonaggio et al., 2020 (99))	161	Δ NLR	Not provided	Baseline and on-treatment	Pts with increase NLR at 6w had worse PFS (HR: 2.2, p<0.0001) and OS (HR: 2.1, p=0.005)	Retro	ΔNLR > 0	Including 86 RCC and 75 NSCLC
(Song et al., 2020 (100))	63	NLR PLR	Pembro, n=42 Nivo, n=4 Sinti, n=17	Baseline	Pts with high NLR had worse OS (HR: 3.14, p=0.004) in multivariate analysis PLR was not significant for OS prediction	Prosp	NLR ≥ 4 PLR > 220	
(Mezquita et al., 2018 (101))	466	dNLR	Not provided	Baseline	Pts with high dNLR had independently worse OS (HR: 1.98, p=0.002)	Retro	dNLR ≥ 3	
(Seban et al., 2020 (102))	63	dNLR	Pembro	Baseline	Pts with high dNLR had independently worse PFS (HR: 2.00, p=0.04) and OS (HR: 3.4, p=0.01)	Retro	dNLR ≥ 3	
(Prelaj et al., 2020 (103))	154	NLR dNLR LMR ΔNLR	Nivo or Pembro	Baseline and on-treatment	Pts with high NLR (HR: 2.59, p<0.001) or dNLR at baseline (HR: 2.20, p<0.001) had worse OS Pts with low LMR had longer OS (HR: 0.45, p<0.001)	Retro	NLR ≥ 4 ΔNLR ≥ 30% dNLR ≥ 2.2 LMR ≥ 1.8	

ICI, Immune checkpoints inhibitors; ALC, Absolut leucocytes count; ANC, Absolut neutrophils count; Nivo, nivolumab; w, weeks; OS, Overall survival; Retro, retrospective; NLR, neutrophil to lymphocyte ratio; Pts, patients; mths, months; PFS, Progression Free-survival; AMC, Absolute monocyte count; PLR, Platelet to lymphocyte ratio; Prosp, prospective; HR, Hazard ratio; ORR, Objective response rate; SII, Systemic Immune-inflammation index; NCP, Neutrophil count percentages; AEC, Absolut eosinophils count; Pembro, Pembrolizumab; WBC, White blood count; PLT, Absolute platelet count; CT, Chemotherapy; PS, Performance status; Toripa, Toripalimab; Sinti, Sintilimab; TPS, Tumor proportion score; LMR, Lymphocyte-monocyte ratio; Durva, Durvalumab; Atezo, Atezolizumab; Camre, Camrelizumab; Tisle, Tislelizumab; RCC, Renal cell carcinoma; NSCLC, Non-small cell carcinoma; n, effective.

Derived neutrophil to lymphocyte ratio (dNLR), assessed by ANC/(WBC – ANC) was also proposed as a promising predictor of ICI responses in NSCLC. We thus reviewed 8 studies exploring dNLR, the largest one enrolling 466 patients (composed of a test set (n=161) and a validation set (n=305)) (101). The patients with high dNLR had independently worse OS (HR: 1.98, p=0.002) while PD-L1 was not significantly predictive of the clinical issue under the ICI regimen. All other studies were consistent and integrated into a meta-analysis published by Yang et al.: the pooled results supported that high dNLR predicted worse PFS (HR: 1.38, p<0.001) and shorter OS (HR: 1.65, p< 0.001) (104). Cut-off values were also different, ranging from 2.2 to 3.0. This meta-analysis showed that dNLR relevance remained significant, indifferently from the dNLR threshold. Moreover, dNLR was commonly associated with a parameter of a global score named LIPI, a predictor of ICI responses, and combined with LDH level. Just as exposed for NLR, the level of evidence is high for LIPI and dNLR but is not integrated into current clinical guidelines for ICI guidance in NSCLC management.

Although many other studies corroborated these observations, this easy and low-cost parameter remains unused in current clinical practice and does not even appear in clinical guidelines.

### 3.6 Comparisons of NLR and PLR respective interests

Petrova et al. explored both NLR and PLR in a cohort, comparing chemotherapy and ICI groups. Both NLR and PLR at baseline were significative predictors of OS in the chemotherapy groups (HR: 8.09, p<0.001 and HR: 2.91, p=0.025, respectively) and ICI groups (HR: 7.94, p<0.001 and HR: 5.08, p<0.001, respectively) in multivariate analysis. This suggests that nor NLR nor PLR are specific for survival in NSCLC treated by ICI (95). Regarding PFS, only NLR remained significant (HR = 4.47, p < 0.001), supporting more interest in NLR parameters in the ICI context.

### 3.7 Limitations and perspectives

Very few studies investigated these blood parameters for patients treated by ICI combined with chemotherapy, whereas chemo-immunotherapy became a large standard for many patients, especially when PD-L1 TPS ≤ 49% in the frontline. Moreover, the inclusion of ICI alone, mainly in a pre-treated context, is not easily transposed in a frontline context which represents a current challenge to predicting patient outcomes. The next step for these potential markers of ICI response remains certainly the establishment of a relevant cut-off to then validate the biological–driven decision in a prospective study.

## 4 Soluble-derived immune checkpoints related products

Although PD-1/PD-L1 were described as membrane-associated molecules, various soluble derived products of ICI were described in the serum of cancer patients. Soluble PD-1/PD-L1 (sPD-1/sPD-L1) and exosomal PD-L1 (exoPD-L1) are both parts of the dynamic PD-1 pathway and immune response (105, 106). Their respective biological effects remain largely unknown. sPD-1 has been proposed to act as a decoy, blocking PD-1 immunosuppressive axis, and binding to PD-L1 and PD-L2 (107–109). sPD-L1 detection and/or high levels could thus be associated with ICI ineffectiveness. Although sPD-L1 effects are not elucidated, its clinical relevance in NSCLC was explored, especially in patients treated by ICI as a predictive biomarker for response and/or tumor progression.

### 4.1 Soluble programmed death ligand 1

sPD-L1 was the most investigated parameter with 9 reviewed studies focusing on ICI outcomes in NSCLC patients including a cohort of 119 NSCLC patients, with a control group of 29 healthy volunteers (110). Additional circulating parameters were explored such as PD-L1 levels on circulating immune cells, platelets, and platelet microparticles. Interestingly, circulating PD-L1+ leukocytes count was independent of tumor PD-L1 expression. Although some features such as PD-L1+ neutrophil count, or PD-L1+ PLTs count were associated with shorter PFS and OS, no differences were observed for patients with high vs low sPD-L1 (cut-off=12.94 pg/ml). Another study equally dimensioned with 233 NSCLC patients treated with Nivolumab or Pembrolizumab (details not provided), reported positive results (111): the patients with high sPD-L1 exhibited both shorter PFS (median: 57 days vs 177 days; p=0.011) and OS (median: 182 days vs not reached, p<0.001) than those in the low sPD-L1 group. In comparison with PD-L1 tissue expression, there was a significant but low correlation between tissue PD‐L1 TPS and circulating sPD‐L1 concentration (r=0.214, p=0.001). sPD-L1 remained an independent predictor of ICI outcome in a multivariate analysis for both PFS (HR: 1.910; p=0.061) and OS (HR: 2.073; p=0.034). In addition, sPD-L1 remained significant in a multivariate analysis for OS prediction after adjustment on PD-L1 tissue expression, illustrating its independence and valuable interest as a predictive marker of ICI benefit. In this report, the discrepancy could be explained by a high threshold of 90 pg/mL. Most other studies examined smaller cohort of patients resulting in lower statistical power and/or considered different cut-offs for sPD-L1 with a large range from 3.357 ng/mL (detection threshold/positivity) to 166 pg/mL (112). Δ sPD-L1 was also investigated with lower interest, as reviewed in **Table 2**. Finally, a meta-analysis updated in 2022 of 710 patients treated by surgery or ICI reported that high levels of sPD-L1 were correlated with worse OS (HR: 2.34; p<0.001) and PFS (HR: 2.35; p<0.001). The results were consistent when focusing on subgroups of patients treated by ICI for OS (HR: 2.40;p<0.001) (124, 125).

**Table 2:** Soluble-derived immune checkpoints-related products.

Study	n	Biomarker	ICI used	Time of assessment	Conclusion	Design	Respective cut-off (If significant)	Comment
(Mazzaschi et al., 2020 (113))	109	sPD-L1	Nivo, n=66, Pembro, n=21, Atezo, n=22	Baseline	Pts with low sPD-L1 had a better PFS (median: 11.9 *vs* 3.8 mths (HR: 2.55; p < 0.001) and OS (median: 15.0 *vs* 5.8 (HR: 2.53; p = 0.001)	Prosp	113 pg/ml	
(Zamora Atenza et al., 2022 (110))	118	sPD-L1	Not provided	Baseline	No differences were observed for pts with high *vs* low sPD-L1	Prosp	12.94 pg/ml	
(Oh et al., 2021 (114))	128	sPD−L1	Nivo, n=41 Pembro, n=32 Durva, n=15, Ipi, n=5, Atezo, n=4	Baseline and on-treatment	High sPD−L1 was associated with worse PFS (median: 2.9 mths *vs* 6.3 mths; p = 0.023), and OS (median: 7.4 mths *vs* 13.3 mths; p = 0.005) In multivariate analyses, high sPD−L1 was an independent prognostic factor for both decreased PFS (HR: 1.928, p = 0.038) and OS (HR: 1.788, p = 0.004).	Prosp	11 pg/μL	128 pts including 50 NSCLC
(Murakami et al., 2020 (111))	233	sPD−L1	Nivo or pembro	Baseline	For high sPD-L1 pts, both PFS (median: 57 d *vs* 177 d; p = 0.011) and OS (median: 182 d *vs* not reached, p < 0.001) were worse than those in the low sPDL1 group sPD-L1 was independently associated with a shorter PFS (HR: 1.910; P = 0.061) and OS (HR: 2.073; P = 0.034) in multivariate analysis.	Retro	90 pg/mL	
(Okuma et al., 2018 (115))	39	sPD-L1	Nivo	Baseline	DCR was better for pts with low plasma sPD-L1 levels (59% vs 25%) Pts with high sPD-L1 levels had a significantly shorter TTF (5.36 mths *vs* 1.48 mths; p = 0.032) and OS (7.20 mths *vs* not reached; p = 0.040)	Prosp	3.357 ng/mL	
(Ando et al., 2019 (116))	21	sPD-L1	Nivo, n=5, Pembro, n=7	Baseline and on-treatment	Reduction sPD-L1 was significantly correlated with tumor regression in pts administered 4 cycles of treatment (p < 0.05). Baseline sPD-L1 in pts who received ICIs were not correlated with the OS	Prosp	Not provided	Same cohort as Ohkuma et al. Focusing on sPD-1 and sPD-L1 alternatively
(Castello et al., 2020 (117))	20	sPD−L1	Nivo, n=12 Pembro, n=7 Nivo+Ipi, n=1	Baseline and on-treatment	No difference in survival outcomes was observed between low sPD-L1 and high sPD-L1 pts An increase in sPD-L1 concentrations during ICI treatment may reflect the expansion of tumor volume and the tumor lysis.	Prosp	27.22 pg/mL	
(Costantini et al., 2018 (118))	43	sPD-L1 sPD-L2	Nivo	Baseline and on-treatment	Baseline sPD-L1 was not associated with ORR High sPD-L1 at 2 mths and increase of sPD-L1 were associated with worse PFS (median: 11.8 mths *vs* 2.2 mths; p = 0.041) with a similar trend for OS (p = 0.087) Pts with an increase sPD−L1 had worse PFS (median: 1.8 mths *vs* 6.5 mths; p = 0.008) and OS (median: 5.4 mths *vs* not reached; p = 0.028) An increase of sPD-L1 remained an independent negative factor for PFS (HR: 4.85; p = 0.048) but not for OS	Prosp	33.97 pg/mL Δ sPD−L1	sPD-L2 did not affect clinical outcomes
(Tiako Meyo et al., 2020 (119))	51	sPD−L1 sPD-1	Nivo	Baseline and on-treatment	sCombo positivity was associated with shorter PFS (median: 78 d *vs* 658 d; HR: 4.12; p = 0.0002) and OS (HR: 3.99; p = 0.003)	Retro	Composite criteria sCombo (limit of détection: 0.156 ng/mL)	Baseline sPD-1 and sPD-L1 were positive for 15 (29.4%) and 27(52.9%) pts, respectively
(Lambert et al., 2022 (120))	40	sPD-1	Budiga	Baseline	Pts with high sPD-1 had better PFS (HR: 0.209; p = 0.002)	Prosp	Not provided	40 NSCLC among 81 pts (41 HNSCC)
(Ohkuma et al., 2021 (121))	21	sPD-1	Nivo, n=5, Pembro, = 7	Baseline and on-treatment	No significant associations between sPD-1 and PFS/OS at baseline or after 2 and 4 cycles of ICI An increased rate of change in plasma sPD-1 concentrations after 2 and 4 cycles of ICI significantly correlated with tumor progression (p = 0.024).	Prosp	Not provided	Small effective including only 12 NSCLC
(Zhang et al., 2020, p. 28 (122))	24	exoPD−L1	Not provided	Baseline	Pts with low exosomal PD-L1 had a better PFS (median: 2.0 mths vs 8.0 mths; p = 0.010)	Prosp	149 pg/mL	
(Shimada et al., 2021 (112))	17	exoPD−L1	Nivo, n= 6, Pembro, n = 11	Baseline	The DCR of 100% for pts with high exosomal PD−L1 (n = 11/17) Pts with high exosomal PD−L1 tended to have a worse RFS in all stages (p = 0.163)	Prosp	166 pg/mL	17 reccurence treated by ICI among 120 pts with stage I–III NSCLC
(Yang et al., 2021 (123))	51	PD-L1 mRNA exoPD-L1 sPD−L1 Δ sPD−L1	Not provided	Baseline and on-treatment	Pts with a fold change of PD-L1 mRNA ≥ 2.04 had better PFS, OS, and bORR A fold change of exoPD-L1 ≥ 1.86 was also associated with better PFS and OS Dynamic change of sPD-L1 was not associated with PFS and OS.	Prosp	Δ PD-L1 mRNA (fold change ≥ 2.04) Δ exoPD-L1(fold change ≥ 1.86)	51 pts including 41 NSCLC

ICI, Immune checkpoints inhibitors; sPD-L1, soluble Programmed Death Ligand 1; Nivo, nivolumab; Pembro, Pembrolizumab; Atezo, Atezolizumab; Pts, patients; PFS, Progression Free-survival; mths, months; HR, Hazard ratio; OS, Overall survival; Prosp, prospective; exo PD-L1, exosomal Programmed Death Ligand 1; sPD-1, soluble Programmed Death 1; Budiga, Budigalimab; NSCLC, Non-small cell carcinoma; HNSCC, Head and Neck squamous cell carcinoma; DCR, Disease control rate; RFS, Recurrence-free survival; TTF, Time to treatment failure; Durva, Durvalumab; Ipi, Ipilimumab; d, days; Retro, retrospective; sCombo, composite criteria (sCombo) corresponding to sPD-1 and/or sPD-L1 positivity for each patient; mRNA, messenger RNA; sPD-L2, soluble Programmed Death Ligand 2; n, effective.

### 4.2 Soluble programmed death ligand 2 and other related parameters

sPD-L2 may act as a decoy blocking the PD-1 immunosuppressive axis and leading to potential ICI inefficacy. Only one study explored the potential interest of sPD-L2 as a predictor of ICI response among 43 NSCLC patients treated with Nivolumab without any association with clinical outcomes (118, 125) (118, 125) (126). One study assessed serum mRNA PD-L1, reporting that patients with a fold change of PD-L1 mRNA ≥ 2.04 had better PFS, OS, and best objective response (123) (**Table 2**). Three studies also explored exoPD-L1, the largest cohort enrolling 42 NSCLC patients with contradictory results. Many other soluble markers might be of interest in this context such as sPD-L2, sLAG3, sTIM-3, or sIDO despite restricted reports (127).

### 4.3 Limitations and perspectives

Thus, all available studies currently suffer from limited effective with lower statistical power and/or retrospective designs. Although sPD-L1 was the most explored parameter with consistent meta-analysis, a relevant cut-off is not defined (high variation across publications). Considering sPD-L1 as a biomarker remains challenging according to its multi-biological and structural protein forms. Other parameters such as exosomal and mRNA-derived products introduced high challenges with technical difficulties, and high variabilities in methodologies, resulting in complex clinical applications.

## 5 Circulating tumor cells

Circulating tumor cells (CTCs) emerged as promising blood based-biomarkers in a large panel of solid cancers (128). CTCs are tumor cells that escaped from the primary tumor site and have extravasated into the blood circulation. CTCs detection is challenging because of their rarety. Nonetheless, recent technologies succeeded in extracting CTCs through enrichment and detection methods based on molecular markers and more especially on epithelial cell adhesion molecules but also on physical parameters (128). CTCs are thus also examined in lung cancer, especially in NSCLC, from early to advanced stages as a predictor of clinical outcomes (17). CTCs harbor a great interest in the cancer context with many potential clinical applications such as early diagnosis markers, prognostic evaluation, therapeutic response monitoring, drug sensitivity testing, and/or precision medication guidance (128, 129). Focusing on the NSCLC context, several studies already reported a global poor prognosis in patients with CTCs detection and/or high CTC enumeration (130), including early and resected NSCLC (131). Here, published studies are reviewed about CTC-related features associated with outcomes under ICI regimens for NSCLC patients.

### 5.1 CTC count

CTC detection and absolute count were the most investigated parameter in the CTC field (132) with at least 8 studies focused on CTC enumeration with ICI treatment. The largest cohort included 104 patients, mainly treated with Nivolumab (133). Based on the CellSearch method, CTC detection was an independent predictive factor for worse PFS and OS at baseline (OR: 0.28, p=0.02), and on-treatment (OR: 0.07, p<0.01). However, this difference did not remain significant after adjustment with other co-factors including PD-L1 TPS (OR:0.22, p=0.08). Another published cohort by Guibert et al. (134) enrolled 96 patients with NSCLC all treated with Nivolumab. The patients with a high baseline CTC number ≥ 30/10mL were associated with worse OS (HR: 1.06; p=0.03) and PFS (HR: 1.05; p=0.02). All others were broadly consistent with these results, whereas we did not identify any meta-analysis focusing on CTC utility with ICI treatment. As reviewed in **Table 3**, the CTC cut-off also varies both on absolute number (0–30) and blood volume collected (3-10 mL), therefore introducing biases.

**Table 3:** Circulating tumor cells as biomarkers.

Study	n	Biomarker	ICI used	Time of assessment	Conclusion	Device	Respective cut-off (If significant)	Comment
(Alama et al., 2019 (135))	89	CTC count	Nivo	Baseline and on-treatment	Pts with CTC < 2/3 mL had better OS (median: 8.8 *vs* 6.2 mths, p = 0.05)	ScreenCell	CTC count ≥ 2/3 mL	Progressing pts with concomitant lower CTCs and cfDNA performed clinically well (p = 0.007)
(Park et al., 2021 (136))	83	CTC count	Pembro, n=18 Atezo, n=65	Baseline and on-treatment	Pts with decreased CTC count from C1 to C2 had better PFS (median: 6.7 *vs* 2.3 mths; p = 0.078) and OS (median: NR *vs* 6.8 mths, p = 0.021)	CD-PRIME	CTC count ≥ 4.6/7.5 mL	CTC count at baseline did not predict ICI response
(Tamminga et al., 2019 (133))	104	CTC count	Nivo, n=89 Pembro, n=8 Atezo, n=5 Nivo+Ipi, n=2	Baseline and on-treatment	CTC detection was an independent predictive factor for worse PFS and OS at baseline (OR: 0.28, p = 0.02), and on-treatment (OR: 0.07, p < 0.01),	CellSearch	CTC count > 0/7.5 mL	
(Mondelo-Macía et al., 2021 (137))	50	CTC count and PD-L1 expression	Pembro	Baseline and on-treatment	Pts with detectable CTC by CellSearch had shorter PFS (median: 3 *vs* 12.6 mths, p < 0.05) and OS (median: 4.9 *vs* 21.1 mths, p < 0.05)	CellSearch and Parsortix systems	CTC count > 0/3 mL PD-L1^+^ by CTCs	13 pts treated by combination CT-ICI
(Dall’Olio et al., 2021 (138))	39	CTC count and PD-L1 expression by CTCs	Nivo, Pembro, or Atezo	Baseline	Median OS in pts with PD-L1^-^ CTC was 2.2 mths *vs* 3.7 mths (HR: 0.33, p = 0.019) in pts with PD-L1^+^ CTC *vs* 16.0 mths, (HR: 0.17 p < 0.001) in pts with no CTC	CellSearch	Divided into 3 groups: no CTC (n = 15), PD-L1^+^ CTC (n = 13), and PD-L1^-^ CTC (n = 11)	No correlation was found between PD-L1 expression by CTCs and tumor tissue CTC number was correlated with baseline tumor size
(Guibert et al., 2018 (134))	96	CTC count and PD-L1 expression	Nivo	Baseline and on-treatment	Baseline high CTC number was associated with worse OS (HR: 1.06; p = .03) and PFS (HR: 1.05; p = .02) Higher baseline PD-L1^+^ CTC number (≥1%) was observed in the “non-responders” group (PFS < 6 mths) (p = .04)	ISET	CTC count ≥ 30/10mL PD-L1^+^ by CTCs (≥1%)	CTCs were more frequently found to be PD-L1 positive than tissue (83% vs 41%) No correlation between tissue and CTC PD-L1 expression (r = 0.04, p = 0.77). PD-L1^+^ CTCs were seen in all pts at progression.
(Dhar et al., 2018 (139))	22	CTC count and PD-L1 expression by CTCs	Pembro, n=10 Nivo, n=2 Nivo+Ipi, n=8 Ave, n=2	Baseline	Not significant	Vortex	CTC count ≥ 1.32/mL ≥ 2 PD-L1^+^ CTCs (overall count)	Analyses were restricted to pts with blood collection immediately before starting ICI (n = 17)
(Ikeda et al., 2021 (140))	45	PD-L1 expression by CTCs	Nivo	Baseline and on-treatment	PFS was significantly longer in pts with ≥ 7.7% PD-L1^+^ CTCs rate (n = 8) than in those with < 7.7% rates (n = 8; p < 0.01) at 8w	MCA system	≥ 7.7% PD-L1^+^ CTCs rate	The cut-off value of the PD-L1 positivity rates in the CTCs was calculated at 4,8 or 12w based on the ROC method
(Nicolazzo et al., 2016 (141))	24	PD-L1 expression by CTCs	Nivo	Baseline and on-treatment	Pts with PD-L1^-^ CTCs all obtained a clinical benefit, while pts with PD-L1^+^ CTCs all experienced progressive disease	CellSearch	PD-L1^+^ by CTCs	Although CTCs were found in all pts 6 mths after treatment, pts could be dichotomized into 2 groups based on PD-L1 expression by CTCs
(Bao et al., 2018 (142))	15	PD-L1, CEA, and hTERT expressions by CTCs	Nivo	Baseline	High expression of CEA (p = 0.017) and hTERT (p = 0.072) by CTCs were associated with poor clinical response	Polymeric microfluid CTC chip	Not provided	Only 2/17 cases had CTCs expressing PD-L1
(Papadaki et al., 2020 (143))	15	CTC count and IDO expression by CTCs	Anti-PD-1	Baseline	Detection CTCs by either ISET (median: 2.5 *vs* 5.8 mths; p = 0.037), Parsortix (median: 2.5 *vs* 6.2 mths; p = 0.036), or any method (median: 2.5 *vs* 10.6 mths; p = 0.007) was correlated with worse PFS IDO^+^ CTCs were associated with shorter PFS (median: 2.5 *vs* 5.8 mths, p = 0.039) and OS (HR: 5.46, p = 0.021)	Parsotix ISET Ficoll	CTC count > 0/5 mL ≥ 1 IDO^+^ CTC (overall count)	Ficoll, ISET, and Parsortix presented the highest yields and compatibility with phenotypic analysis At the pts level, they provided discordant CTC positivity (13%, 33%, and 60% of pts, respectively) PD-L1 was expressed in 33% of CTCs

ICI, Immune checkpoints inhibitors; CTC(s), Circulating tumor cells; Nivo, nivolumab; OS, Overall survival; mths, months; cfDNA, Cell-free DNA; Pts, patients; PD-L1, Programmed death ligand 1; Pembro, Pembrolizumab; PFS, Progression Free-survival; CT, Chemotherapy; ISET, Isolation by SizE of Tumor cells; Atezo, Atezolizumab; Ipi, Ipilimumab; Ave, Avelumab; CEA, Carcinoembryonic antigen; hTERT, human Telomerase reverse transcriptase; IDO, indoleamine-2,3-dioxygenase 1; PD-1, Programmed death 1; HR, Hazard ratio; w, weeks; MCA, automated microcavity array; ROC, Receiver Operating Characteristic; n, effective.

### 5.2 PD-L1 expression by CTC

Additional techniques and analyses have been proposed to improve CTC relevance in the ICI context besides CTC detection and enumeration. Among them, PD-L1 expression by CTC was most explored (7 studies) with the hypothesis that PD-L1 expression by CTCs might be a valuable surrogate for PD-L1 tissue expression, in a dynamic and non-invasive approach, representing the whole landscape of the tumor heterogeneity. The largest study observed a higher baseline PD-L1+ CTC number (≥1%) in the “non-responders” group (PFS < 6 months, p=0.04) whereas PD-L1 TPS did not manage to predict ICI benefit in terms of PFS (134). No correlation was observed between tissues and CTC PD-L1 expression (r=0.04, p=0.77), and CTCs were more frequently found to be PD-L1 positive than tissues (83% vs 41%). Finally, PD-L1+ CTCs were seen in all patients at progression. Mondelo-Macía et al. reported no association for PD-L1+ CTCs, regardless of the technology employed (CellSearch and Parsortix systems) (137). A recent meta-analysis of 30 studies including various cancers reported global prognostic factors associated with PD-L1 CTC expression (144). Furthermore, Ouyang et al. reported that the baseline presence of PD-L1+ CTC was associated with better PFS (HR: 0.55, p=0.084, I2 = 61.1%, p=0.025) and with a trend for OS (HR: 0.61, p=0.067, I2 = 43%, p=0.135) when treated by ICI. In contrast, non-immune-based treatment (chemotherapy and/or TKI) was associated with worse PFS (HR: 1.85, p=0.005, I2 = 60.6%, p<0.001) and OS (HR: 2.44, p<0.001, I2 = 42.2%, p<0.043). These results suggested that PD-L1 CTC expression could endorse a certain specificity to predict outcomes with ICI treatment. However, PD-L1 CTC expression was not a significant predictor of PFS in the NSCLC context (HR: 1.3, p=0.341, I2 = 58.0%, p=0.011) with similar data for OS prediction.

### 5.3 Limitations and perspectives

CTCs seem highly promising for clinical cancer management, based on their non-invasive, easily repeatable, and dynamic real-time monitoring analysis (145). Additional CTC markers have been explored in NSCLC patients treated by ICI. In a cohort of 15 patients, high Carcinoembryonic antigen (CEA) and Human telomerase reverse transcriptase (hTERT) expression on CTC were associated with poor clinical response (p=0.017 and p=0.072, respectively) (142). In an equally dimensioned group treated by ICI, Indoleamine 2,3-dioxygenase (IDO)+ CTC detection was associated with shorter PFS (median: 2.5 vs 5.8 months, p=0.039) and OS (HR: 5.46, p=0.021). However, these results remain exploratory.

CTC use and transposability in daily practice remain very challenging. CTC detection remains very challenging with so-far only two FDA-approved methods (CellSearch and Parsortix) for specific cancer contexts and other numerous non-standardized techniques including ISET^®^ (Isolation by Size of Epithelial Tumor cells) or numerous microfluidic systems. CellSearch is an FDA-approved system that demonstrated its clinical relevance in other solid cancer including breast (146), colorectal (147), and prostate cancers (148). The CellSearch method enriches cells using a magnetic ferrofluid containing antibodies against epithelial cell adhesion molecules (i.e EpCAM), before staining for cytokeratins (including cytokeratins 8, 18, and 19). This technology is not included in clinical practice, most probably because of its high cost. Moreover, there is no consensus for a relevant CTC threshold, also depending on the volume of blood collected. This critical point requires further investigation before additional consideration by the clinician. Innovative approaches are emerging such as Circulating tumor-derived endothelial cells (CTECs) that could predict acquired resistance to ICI (149). Other rare types of CTCs are suggested with non-elucidated and incertain clinical relevance (150). Aside from CTC-expressed immune-related biomarkers, other markers are also highly promising in reflecting tumor immune resistance (primary or acquired) such as those acquired through Epithelial-Mesenchymal Transition (EMT) for instance (151).

## 6 Circulating tumor DNA

Plasma circulating tumor DNA (ctDNA) is a cell-free DNA product released by the tumor in the bloodstream. ctDNA interest is growing fast for solid tumor management. The detection and monitoring of ctDNA provide new opportunities for personalized cancer management. ctDNA is already used in clinical practice for detecting some targetable oncogenic driver such as EGFR of BRAF mutation but might have additional interest in the NSCLC context (152). Diverse technologies to analyze plasma ctDNA emerged and progressively integrated clinical practice. However, there is high variability and a lack of standardized techniques to detect ctDNA such as allele-specific PCR, digital PCR, multiplex PCR-based NGS, and whole-exome sequencing (WES) (153). Nonetheless, ctDNA-based clinical decision-making holds significant potential despite challenges and complexities, especially in the field of immunotherapy and lung cancer. We reviewed here published studies on ctDNA-related features associated with outcomes under ICI regimens for NSCLC patients (154).

### 6.1 Cell-free DNA and derived biomarkers

Cell-free DNA can be estimated by various methods, most commonly using the maximum somatic allele frequency (MSAF), which is defined as the maximum allele frequency (AF) of all the tumor somatic mutations observed per sample by next-generation sequencing (NGS), and reflecting the ctDNA proportion in the blood. ctDNA and especially its variation and clearance have also been proposed as a potential surrogate of early tumor response and might predict responses to ICI (12). We identified 18 studies based on cell-free DNA detection and quantification of patients’ outcomes with NSCLC and under ICI (**Table 4**). The largest tested ctDNA levels as a potential relevant surrogate of early tumor response to ICI (168). ctDNA was detected using non-targetable mutation from the initial tumor biopsy by droplet digital PCR. A ctDNA decrease of over 30% at 4-6 weeks was correlated with an improved PFS and OS in 100 AC treated by ICI. In this cohort, patients with a tissue-positive PD‐L1 expression (TPS ≥ 1%) had a better PFS (HR: 0.46, p<0.001) and OS (HR: 0.57, p<0.05) than PD‐L1 negative patients. However, ctDNA demonstrated its independency from PD-L1 expression, significantly predicting OS both in PD-L1 positive and negative patients (HR: 0.37 and 0.47, p<0.05; respectively). Another equally dimensioned cohort of 97 patients treated by ICI also correlated with patient outcomes: an increase of ctDNA allele fraction at 1 month was associated with a 2-month PFS versus 14 months for patients with a decrease of AF. On another hand, PD-L1 TPS was not statistically predictive of ICI benefit, using either a cut-off of 1% or 50%, and was less predictive of response than ctDNA profiling (165). The largest report based on ctDNA was a pooled analysis of the randomized POPLAR and OAK studies by Chen et al. (161). This study assessed the clinical relevance of maximum somatic allele frequency (MSAF) which is an indicator of the proportion of tumor-derived plasma DNA. Atezolizumab was identified as beneficial when patients harbored lower MSAF levels (i.e., MSAF < 10.3%; HR: 0.59, p<0.001). In contrast, no difference was observed for patients with high MSAF levels between docetaxel and ICI groups (HR: 0.91, p=0.5). In this analysis, subgroup comparisons were performed especially regarding clinical confounding factors to determine the independency of ctDNA. Thus, the prediction of ICI interest by ctDNA remains significant both in Atezolizumab and Docetaxel arms after adjustment for baseline covariate (including age, sex, race, performance status, histology, number of metastatic sites, smoking history or number of prior therapies) with a more prominent effect in the Atezolizumab arm (HR=1.89, p<0.001 for Atezolizumab vs. HR=1.30, p=0.029 for docetaxel group). Finally, a meta-analysis was performed on 10 studies, including 1017 patients with NSCLC and treated by ICI (169). The baseline ctDNA detection was not associated with clinical outcomes, for OS, PFS, and ORR (respective HR: 1.18; 0.98, and 0.89). The longitudinal assessment and especially its early decrease was able to significantly predict ICI benefit regarding both OS, PFS, and ORR (respective HR: 0.19, 0.30, and 0.07).

**Table 4:** Circulating tumor DNA as a biomarker.

Study	n	Biomarker	ICI used	Time of assessment	Conclusion	Device	Design	Respective cut-off (If significant)	Comment
(Thompson et al., 2021 (155))	67	ctDNA VAF	Pembro+/-CT	Baseline and on-treatment	Molecular responders had significantly longer PFS (HR: 0.25) and OS (HR: 0.27)	74-gene NGS panel	Prosp	Δ ctDNA < 50%	
(Chen et al., 2020 (156))	22	ctDNA VAF	Camrelizumab and anti-angiogenic (Apatinib)	Baseline	High concentration of cfDNA (HR: 27.7, P = 0.003), MIKI67 mutation (HR: 114.1, p = 0.009), and gene variations related to hyper-progressive disease (HPD) (HR: 36.8, p = 0.004) were independent risk factors for worse PFS	dsDNA HS assay kit	Prosp	ctDNA detectable	
(Hellmann et al., 2020 (157))	31	ctDNA VAF	Not provided	Baseline and on-treatment	27 pts had undetectable ctDNA and 25 (93%) have remained PF all 4 pts with detectable ctDNA progressed (p < 0.0001; PPV= 100%, NPV = 93%)	Deep Sequencing (CAPPSeq)	Retro	ctDNA detectable	
(Raja et al., 2018 (158))	100	ctDNA VAF	Pembro	Baseline and on-treatment	In the validation NSCLC cohort, the mean VAF decreased by 4% (p = 0.0009) in pts with CR/PR and 1.1% (p = 0.02) in pts with SD, whereas the mean VAF increased by 1.4% (p = 0.03) in pts with PD	Guardant360	Prosp	Δ VAF ≥ 0%	Included only 28 NSCLC
(Passiglia et al., 2019 (77))	45	ctDNA	Nivo	Baseline and on-treatment	Pts with increased cfDNA >20% at the 6^th^ w reported significantly worse outcomes (median OS: 5.7 vs 14.2 mths, p < 0.001; median PFS: 3.3 vs 10.2 mths, p < 0.001)	dsDNA HS assay kit	Retro	Δ ctDNA >20% at 6 w	
(Alama et al., 2019 (135))	89	ctDNA	Nivo	Baseline and on-treatment	Pts with baseline CTC number and cfDNA below their median values (2 from 3 mL and 836.5 ng from 3 mL of blood and plasma, respectively) survived significantly longer than those with higher values (p = 0.05 and p = 0.04, respectively)	qPCR using hTERT as a reference	Prosp	> 836.5 ng cfDNA/3 mL of plasma	
(Anagnostou et al., 2019 (159))	38	ctDNA	Nivo, n=28 Pembro, n=8 Others, n=2	Baseline and on-treatment	Pts without a molecular response had shorter PFS and OS compared with molecular responders (median: 5.2 vs. 14.5 and 8.4 vs. 18.7 mths; HR: 5.36, p = 0.007 and HR: 6.91, p = 0.02, respectively)	58-gene NGS panel	Prosp	Δ ctDNA < 0%	Pts with acquired resistance had a recrudescence in ctDNA levels
(Li et al., 2019 (160))	12	ctDNA MSAF	Pembro	Baseline and on-treatment	MSAF in the SD/PD group was significantly higher than in the PR group (p = 0.00044).	329-gene NGS panel	Prosp	N/A	Included 10 Sq-NSCLC and 4 in L2+
(Chen et al., 2019 (161))	853	ctDNA MSAF	Atezo	Baseline	OS was significantly improved with ICI compared with CT in those with low MSAF (HR: 0.57, p < 0.001)	F1CDx	Retro	MSAF<10.3%	Meta-analysis of POPLAR and OAK studies
(Goldberg et al., 2018 (162))	28	ctDNA AF	Not provided	Baseline and on-treatment	A ctDNA decrease was associated with superior PFS (HR: 0.29, p = 0.03) and OS (HR: 0.17, p = 0.007)	24-gene NGS panel	Prosp	Δ AF ≥ 50%	Low effective
(Iijima et al., 2017 (163))	14	ctDNA AF	Nivo	Baseline and on-treatment	Initial and serial ctDNA analysis revealed that a decrease in AF of ctDNA correlated with durable benefit	53-gene NGS panel	Prosp	AF ≥ 2%	Low effective
(Ricciuti et al., 2021 (126))	62	ctDNA AF	Pembro	Baseline and on-treatment	AF decreases between the baseline and 1^st^ on-treatment blood evaluation was associated with significantly higher ORR (60.7% vs 5.8%, p = 0.0003), and longer PFS (median: 8.3 vs 3.4 mths, HR: 0.29, p = 0.0007) and OS (median: 26.2 vs 13.2 mths, HR: 0.34, p = 0.008)	36-gene NGS panel	Prosp	Δ AF ≥ 0%	
(Giroux Leprieur et al., 2018 (164))	15	ctDNA AF	Nivo	Baseline and on-treatment	Good diagnostic performances for tumor response and clinical benefit, both for: a/ctDNA concentration at the 1^st^ tumor evaluation (tumor response: PPV = 100%, NPV = 71%; clinical benefit: PPV = 83.3%, NPV = 77.8%) b/ctDNA change at the 1^st^ tumor evaluation (tumor response: PPV = 100%, NPV = 62.5%; clinical benefit: PPV = 100%, NPV 80%) Pts with ctDNA concentration increase < 9% at 2 mths had a long-term benefit of ICI	22-gene NGS panel	Prosp	Δ ctDNA > 9%	No difference observed between ctDNA at baseline
(Nicolas Guibert et al., 2019 (165))	97	ctDNA AF	Nivo, n=90 Pembro, n=7	Baseline and on-treatment	Early decreases in the ctDNA AF were associated with longer PFS (median: 14 vs 2 mths, p < 0.0001)	36-gene NGS panel	Retro	Δ AF ≥ 0%	
(Sun et al., 2021 (166))	73	ctDNA Genomic alterations in PTPRs-related genes	Not provided	Baseline	Among all PTPRs, PTPRD mutations in non-Sq NSCLC were linked to longer PFS (median: 324 vs 63 d, HR: 0.36, p = 0.0152) and higher ORR (p = 0.0099).	NGS	Prosp	Mutation presence in at least one PTPRs-related genes	Pooled cohort including OAK and POPLAR trials
(Mondelo-Macía et al., 2021 (137))	50	hTERT ctDNA	Pembro	Baseline and on-treatment	Pts with high baseline hTERT cfDNA levels had significantly shorter PFS and OS than those with low baseline levels Multivariate regression analyses confirmed the relevance of the combination CTCs/cfDNA levels as an early independent predictor for PD	hTERT qPCR	Prosp	hTERT cfDNA > 2132.39 GE/mL	
(Brueckl et al., 2021 (167))	45	ctRNA for CD3, CD8, PD-1, PD-L1, CTLA-4	Pembro	Baseline and on-treatment	An increase in CD3 and CD8 mRNA expression after the 1^st^ cycle of pembrolizumab was associated with improved PFS and OS	rt-qPCR	Retro	Δ CD3 > 0 Δ CD8 > 0	
(van der Leest et al., 2021 (168))	100	ctDNA based on non-targetable mutations present in the tumor biopsy	Nivo, n=69 Pembro, n=28 Atezo, n=2 Durva, = 1	Baseline and on-treatment	A > 30% decrease in cfDNA at t1 correlated with a longer PFS and OS	ddPCR	Prosp	Δ ctDNA > 0	

ICI, Immune checkpoints inhibitors; hTERT, hTERT: human Telomerase reverse transcriptase; ctDNA, circulating tumor DNA; Pembro, Pembrolizumab; Pts, patients; PFS, Progression Free-survival; OS, Overall survival; CTC(s), Circulating tumor cells; PD, Progressive disease; q-PCR, quantitative Polymerase chain reaction; Prosp, prospective; GE, Genome equivalent; CDx, Cluster of differenciation x; PD-L1, Programmed death ligand 1; CTLA-4, cytotoxic T-lymphocyte-associated protein 4; rt, reverse transcriptase; Retro, retrospective; Nivo, nivolumab; Atezo, Atezolizumab; Durva, Durvalumab; ddPCR, droplet digital PCR; PTPRs, protein tyrosine phosphatase receptor-type; Sq, Squamous; NSCLC, Non-small cell carcinoma; HR, Hazard ratio; ORR, Objective response rate; NGS, next-generation sequencing; VAF, Variant allele fraction; CT, Chemotherapy; mths, months; PPV, Positive predictive value; NPV, Negative predictive value; w, weeks; dsDNA, Double stranded DNA; MSAF, Maximum somatic allele frequency; SD, Stable disease; PR, Partial response; L2, 2^nd^ line; CR, Complete response; n, effective.

### 6.2 Blood Tumor mutational burden

Tumor mutation burden (TMB) is a marker of genomic instability. It reflects the production of immunological and inflammatory neoantigens, closely related to immunogenicity (170). The biological approaches support that high TMB reflects a tumor with a higher level of mutation and neoantigens productions, reflecting a so-called “hot-tumor”, which could potentially be predictive of ICI sensibility (171). Previous clinical trials reported positive results from tissue-based TMB analysis in NSCLC (172, 173). Blood TMB (bTMB) is a derived marker of ctDNA that might have the capacity to integrate and reflect tumor heterogeneity, and that we reviewed in a dedicated section (**Table 5**) according to a specific method of assessment and analysis.

**Table 5:** Blood Tumor mutational burden.

Study	n	Biomarker	ICI used	Time of assessment	Conclusion	Device (coverage of genomic regions)	Design	Respective cut-off (If significant)	Comment
(Gandara et al., 2018 (174))	1070	bTMB	Atezo	Baseline	Pts with high bTMB had significantly longer PFS (HR: 0.65, p = .013) from ICI *vs* CT	F1CDx (1.125 Mb)	Retro	bTMB ≥ 4 and > 26	Meta-analysis of POPLAR and OAK studies The bait set targeted 1.125 Mb of the coding region of the human genome
(Wang et al., 2019 (175))	50	bTMB	Not provided	Baseline	bTMB > 6 was associated with superior PFS (HR: 0.39; p = 0.01) and ORR (39.3% *vs* 9.1%; p = 0.02)	NCC-GP150 (0.6 Mb)	Retro	bTMB > 6	
(Wang et al., 2020 (176))	737	LAF-bTMB	Atezo	Baseline	bTMB was not associated with outcomes No correlation owing to its correlation with the amount of circulating tumor DNA	F1CDx (1.125 Mb) NCC-GP150 (0.6 Mb)	Retro	bTMB ≥ 16 bTMB > 6	POPLAR, n =211, and OAK, n = 462 and validated in the 3^rd^ NCC cohort (n = 64)
(Ba et al., 2021 (177))	2338	bTMB	Atezo, Durva +/- Treme, Tisle, Pembro,	Baseline	Compared with CT, ICI improved OS (HR: 0.62; p < 0.01), PFS (HR: 0.57; p < 0.01), and ORR (OR: 2.69; p < 0.01) in bTMB-high NSCLC pts but not in bTMB-low pts	F1CDx Gardant OMNI OncoScreen Plus	Retro	bTMB ≥ 16	Meta-analysis from 6 RCT
(Li et al., 2019 (160))	12	bTMB	Pembro	Baseline and on-treatment	High baseline bTMB significantly improved PFS after ICI (p = 0.048) bTMB at reevaluation was not associated with outcomes	329 pan cancer‐related gene panel (0.637 Mb)	Prosp	bTMB > 21	Included 10 Sq-NSCLC and 4 in L2+
(Chen et al., 2019 (161))	853	bTMB	Atezo	Baseline	OS was significantly improved with ICI *vs* CT in pts with a high bTMB (HR: 0.57, p < 0.001)	F1CDx (1.125 Mb)	Retro	bTMB ≥ 13	Meta-analysis of POPLAR and OAK studies
(Chae et al., 2019 (178))	136	bTMB	Not provided	Baseline	Higher bTMB significantly correlated with shorter PFS (median: 45 *vs* 355 d; HR: 5.6, p < 0.01) and OS (median: 106 d *vs* not reached; HR: 6.0, p < 0.01)	Guardant 360 (0.138 Mb)	Retro	Median value of 14.5, 7.2, and 21.7	Included both the first line and more
(Jiang et al., 2022 (179))	270	bTMB	Camre	Baseline and on-treatment	Baseline bTMB was not associated with ORR, PFS, and OS in Camre or placebo + CT groups Low on-treatment bTMB was associated with better ORR (73.8% *vs* 27.8%, p < 0.001), PFS (median: 9.1 *vs* 4.1 mths, p < 0.001), and OS (median: not reached *vs* 8 mths, p < 0.001) in Camre + CT group	HyperCap Kit (1.6 Mb - 543 cancer-related genes)	Prosp	Defined as bTMB ≥ 75% level (absolute value not provided) Δ bTMB ≥ 0	Only Sq-NSCLC tumors
(Kim et al., 2022 (180))	152	bTMB	Atezo	Baseline	At 36.5-mths follow-up, an exploratory analysis found that bTMB was associated with longer OS bTMB remained associated with higher ORR	F1CDx (1.125 Mb)	Prosp	bTMB ≥ 16	B-F1RST study bTMB was not associated with PFS ORR improved as bTMB cutoffs increased
(Rizvi et al., 2020 (181))	809	bTMB	Durva +/- Treme	Baseline	High bTMB improved OS for Durva + Treme *vs* CT (median: 21.9 mths *vs* 10 mths, HR: 0.49)	F1CDx (1.125 Mb)	Prosp	bTMB ≥ 20	MYSTIC study
(Chen et al., 2021 (182))	56	bTMB	Not provided	Baseline	Pts with high bTMB had better PFS (median: 5.8 mths *vs* 2.0 mths; p = 0.0029)	OncoScreen (based on plasma NGS of 520 genes) (1.64 Mb)	Prosp	bTMB ≥ 11	
(Ma et al., 2021 (183))	70	bTMB	Not provided	Baseline	High bTMB was related to better PFS (HR: 0.32, p < 0.01) and ORR (83.3% *vs* 14.2%, p = 0.02)	F1CDx (1.125 Mb)	Retro	bTMB ≥ 6	

ICI, Immune checkpoints inhibitors; bTMB, Blood-Tumor mutational burden; Atezo, Atezolizumab; Pts, patients; mths, months; PFS, Progression Free-survival; HR, Hazard ratio; CT, Chemotherapy; F1CDx, FoundationOne CDx; Retro, retrospective; ORR, Objective response rate; LAF, Low allele frequency; NCC, National Cancer Center; Durva, Durvalumab; Treme, Tremelimumab; Tisle, Tislelizumab; Pembro, Pembrolizumab; NSCLC, Non-small cell carcinoma; RCT, Randomized controlled trials; Prosp, Prospective; Sq, Squamous; L2, 2^nd^ line; Camre, Camrelizumab; Mb, Megabases; NGS, next-generation sequencing; n, effective.

The largest cohort included 809 patients with a NSCLC treated with Durvalumab +/- Tremelimumab (vs SOC) in the MYSTIC study (181). With a threshold of 20 mutations/Mb at baseline, the patients with high bTMB had better OS (HR: 0.49) when treated with Durvalumab and Tremelimumab. A level of bTMB > 16 mutations/Mb was associated with better OS in 153 patients treated with Atezolizumab from the BF1RST trial (at baseline, HR: 0.54, p<0.5) (180). Gandara et al, compared PD-L1 expression and bTMB levels to assess the potential overlap between these two parameters (174). Among 1070 patients (pooled from OAK and POPLAR trials), patients with high bTMB (>16 mutations/Mb) were not overrepresented among patients with the highest levels of PD-L1 (defined by a TPS ≥50% or ≥10% of tumor-infiltrating immune cells expressing PD-L1) with only 30 patients positive for both assays (19.2% of patients for bTMB and 29.1% for PD-L1 expression). These data demonstrated the independence between bTMB and PD-L1 expressions assessed by IHC. The supplemental analysis from the same publication also compared the clinical characteristics of the bTMB subgroups of patients from the OAK trial. bTMB > 16 mutations/Mb was associated with smoking history as already published, as a consequence of tobacco mutagen exposure (184). Additionally, high bTMB was also associated with high tumor stages (p<0.0001) or the number of metastases sites(p=0.0055), potentially impacting survival and prognosis. However, the baseline clinical features were well balanced between arms (Atezolizumab vs. Docetaxel) suggesting that bTMB might be an independent predictive marker of ICI efficacy.

Only one study addressed the potential relevance of longitudinal bTMB assessment (179): no correlation was found between clinical outcomes and baseline bTMB in 270 lung squamous carcinomas treated by Camrelizumab. However, low on-treatment bTMB significantly correlated with better PFS, OS, and ORR. This association was specific to the ICI regimen and was not observed in the chemotherapy control group. Finally, a meta-analysis pooled 2338 patients from 6 randomized controlled trials with bTMB assessment (177): patients with high bTMB and treated by ICI had improved ORR (HR:2.69, p<0.03), PFS (HGR: 0.57, p<0.01), and OS (HR: 0.62, p<0.01) in comparison with patients treated with chemotherapy. Inversely, no clinical benefit was observed with ICI regimens when patients had lower bTMB. More interestingly, subgroups analyses confirmed across all potential confounding factors (such as line of treatment, type of NGS panel with various among of genome covered, level of PD-L1 expression, and ICI regimen) that bTMB was able to independently predict clinical issues for NSCLC patients treated by ICI.

The interest in soluble biomarkers is also emerging in the earlier stages, including neoadjuvant conditions with many ICI trials in the peri-operative context (185). The NADIM trial enrolled 46 patients with a locally advanced stage IIIA NSCLC treated with neoadjuvant chemotherapy and Nivolumab (186). Tissue-based TMB and PD-L1 were significant predictors of OS. The patients with the lowest ctDNA at baseline had longer PFS (HR: 0.20, p=0.006), and OS (HR: 0.27, p=0.002). Moreover, the absence of ctDNA after neoadjuvant treatment was also associated with improved PFS (HR: 0.26, p=0.038) and OS (HR: 0.04, p=0.015). These results illustrated the feasibility and clinical relevance of detecting soluble biomarkers in every stage of NSCLC treated by ICI. Prospective dedicated studies are further needed to improve clinical outcomes under ICI regimen, considering randomization with pre-specified soluble biomarker expression and/or level at baseline.

### 6.3 Limitations and perspectives

ctDNA and its related biomarkers are highly promising for cancer management. However, many limitations remain unsolved.

The baseline level of ctDNA, and particularly of MSAF could be biased. An MSAF < 1% was associated with better OR in the BF1RST study. However, this result could be driven by better baseline values rather than by MSAF itself (180). Although the findings are broadly consistent, establishing bTMB cut-off values still requires further studies. For instance, the Keynote-189 trial used a 15 mutation/Mb while the MYSTIC study was based on a bTMB threshold of 20 mutation/Mb. Finally, OAK, B-F1RST, and POPLAR trials used an intermediate threshold of 16 mutations/Mb. Moreover, bTMB determination suffers from a major lack of standardization. Gold standard techniques involve a WES examination. Trying to reduce cost and time analysis, clinical studies were based on various NGS panels covering 150 to more than 500 genes and thus covering variable amounts of the genome (from 0.138 to 1.64 Mb of coding exome; the literature recommends at least 1 Mb of DNA for reliable assay) without clear cut-off for bTMB (187, 188).

These major reports highlight the need for a relevant bTMB cut-off before conducting a randomized controlled trial with a biological-driven treatment decision.

## 7 Perspectives for liquid biopsies into the immune landscape

### 7.1 Current active clinical trials

As reviewed previously, many circulating markers might help clinicians to predict ICI outcomes for NSCLC patients (189). We summarized here current active clinical trials investigating one or more soluble biomarkers in the ICI context for NSCLC and discussed how their results could introduce a change into clinical practice.

No active clinical trial is investigating (d)NLR in NSCLC despite its high potential. The BUDDY trial (NCT04059887) explores bTMB as a biomarker, whereas the endpoints do not drastically differ from the BF1RST study previously discussed. The NCT03373955 trial (non-randomized design) aims to construct an immune repertoire for patients treated with Atezolizumab, mainly based on T-cell repertoire and cfDNA. This approach might provide additional promising soluble markers.

The NCT04720339 prospectively enrolls NSCLC patients treated with Atezolizumab, assessing the predictive value for quantification of plasma cfDNA at the time of the first radiological evaluation and on clinical benefit. The ATLAS and CIRCULAR trials have similar secondary endpoints, based on Nivolumab-Ipilimumab or Pembrolizumab regimen (NCT04966676 and NCT04912687, respectively). Complementary results from observational studies are also expected with the same scope (NCT03892096; NCT04791215).

The COPE trial is an ambitious biological-driven protocol, where implementing sequential ctDNA to improve the management of patients with advanced cancer and therefore their survival is tested. AstraZeneca also supports a recruiting trial assessing the benefit of adjuvant concomitant chemotherapy plus Atezolizumab for resected NSCLC patients with post-operative detectable ctDNA (NCT04367311) named molecular residual disease (MRD). The clearance of ctDNA will also serve as a surrogate for DFS and OS. Some trials are also recruiting based on MRD positivity (detectable cfDNA) with other ICI regimens like Durvalumab + Tremelimumab (NCT04625699) also in post-operative context, post stereotactic radiotherapy for stage I (SCION trial - NCT04944173) or Pembrolizumab metastatic frontline (NCT05198154).

The terms of CTC use remain challenging. Many trials aim to use CTC as a surrogate of tumor response in ICI treatment through plasma clearance (NCT05091190; NCT03481101). The largest study dedicated to CTC in NSCLC is currently the IMMUNO-PREDICT trial, aiming to enroll about 200 patients with an NSCLC. Its main objective is to demonstrate the feasibility of the analysis of PD-L1 expression on CTC.

These active trials reflect the need to implement biomarkers into clinical management, especially with ICI treatment and in each NSCLC stage.

### 7.2 Future directions and potential impact on clinical practice

**Figure 2:**
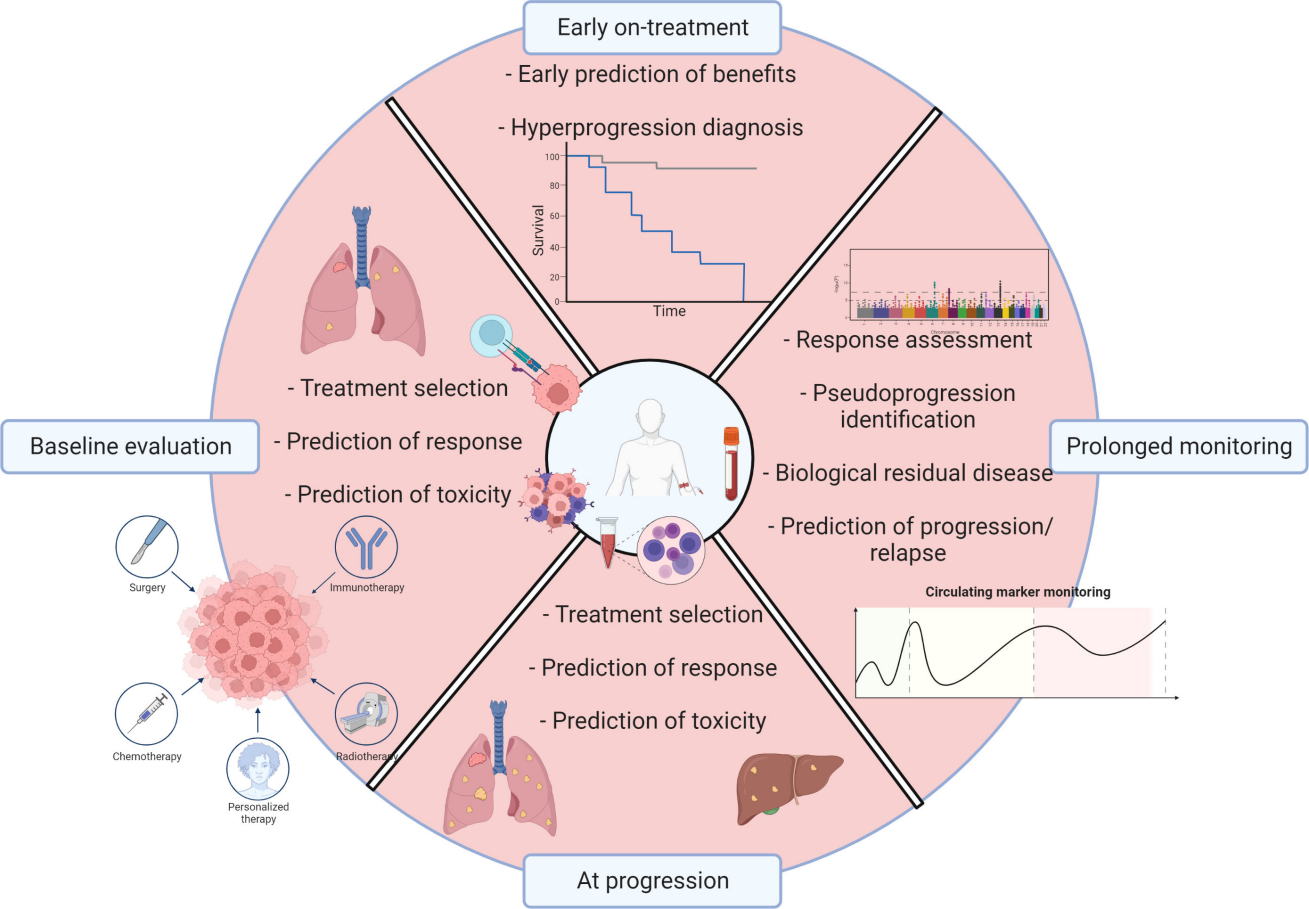
Perspectives for liquid biopsies into the immune landscape. Liquid biopsy for patients with NSCLC and treated by immunotherapy can improve many clinical challenges such as baseline evaluation, real-time monitoring, and prediction of response or progression. Created with BioRender.com.

The experimental strategies do not converge on the same aims, challenges, and difficulties according to the stage of the disease. We discuss here how soluble biomarkers could be integrated into clinical practice with their respective potential interest. At the baseline evaluation, soluble biomarkers could thus allow the selection of the more appropriate treatment with a higher predictive value of clinical benefit (190). It could also predict patients with worse prognoses to propose more aggressive treatment and combine ICI with chemotherapy rather than ICI alone. At the early on-treatment phase, soluble biomarker variations could identify patients with biological progression and predict patients with a higher risk of progression to closely monitor the disease (191), and even identify patients with early-stage hyper progression (192). The prolonged monitoring of soluble biomarkers for patients treated by ICI also exhibits additional interest such as response assessment. The pseudo-progression or prediction of biological residual disease in long-term responders could also be identified with prolonged monitoring of soluble biomarkers (193) (**Figure 2**).

## 8 Conclusion

Recent advances in lung cancer management are particularly impressive in ICI. However, only a sub-population benefits from immunotherapies. The currently available biomarkers, including tumor PD-L1 expression, remain largely perfectible. Liquid biopsy is now well-admitted into NSCLC with oncogenic addiction treated by TKIs showing promising results in the ICI field. Easy-to-use parameters derived from blood numerations and more complex scores and parameters can predict ICI outcomes for patients with NSCLC. However, each parameter harbors various limitations growing roots from a low level of evidence to technical difficulties before the integration into clinical practice. The design of specific and dedicated clinical trials is necessary to improve patient survival with biological-driven randomization and/or management.

## Author contributions

JA, VDo, GD, and MP contributed to the conception and design of the study. JA wrote the first draft of the manuscript. JA, VDo, and VDa wrote sections of the manuscript. All authors contributed to the manuscript revision, and approved the submitted version.
